# Dicarbonyl­dichlorido(*N*,*N*,*N*′,*N*′-tetra­methyl­ethylenediamine)­ruthenium(II)

**DOI:** 10.1107/S1600536811022227

**Published:** 2011-06-18

**Authors:** Ahmad O. Baghlaf, Muhammad Ishaq, Salih S. Al-Juaid, Abdullah M. Asiri, Muhammad Nadeem Arshad

**Affiliations:** a Chemistry Department, Faculty of Science, King Abdul Aziz University, PO Box 80203, Jeddah 21589, Saudi Arabia; b The Center of Excellence for Advanced Materials Research, King Abdul Aziz University, Jeddah, PO Box 80203, Saudi Arabia; c X-ray Diffraction and Crystallography Laboratory, Department of Physics, School of Physical Sciences, University of the Punjab, Quaid-e-Azam Campus, Lahore-54590, Pakistan

## Abstract

In the title compound, [RuCl_2_(C_6_H_16_N_2_)(CO)_2_], the geometry around the Ru^II^ atom is a distorted RuC_2_N_2_Cl_2_ octa­hedron, with pairs of C and Cl atoms *trans* to each other and the N atoms of the bidentate ligand in a *cis* conformation. The five-membered chelate ring is puckered on the C—C bond.

## Related literature

For background to ruthenium carbonyl derivatives, see: Manchot & Konig (1924 ▶); Stephenson & Wilkinson (1966 ▶); Kingston *et al.* (1967 ▶); Baghlaf *et al.* (2007 ▶); Campbell (1975 ▶); Padhey & Kaufman (1985 ▶). For a related structure, see: Bakar *et al.* (1993 ▶).
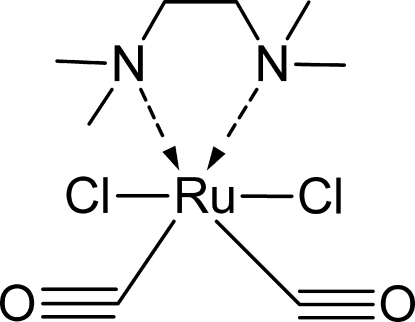


## Experimental

### 

#### Crystal data

[RuCl_2_(C_6_H_16_N_2_)(CO)_2_]
*M* *_r_* = 344.20
Monoclinic, 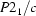

*a* = 7.463 (6) Å
*b* = 14.579 (6) Å
*c* = 12.718 (12) Å
β = 106.37 (8)°
*V* = 1327.7 (17) Å^3^
*Z* = 4
Mo *K*α radiation
μ = 1.57 mm^−1^
*T* = 160 K
0.38 × 0.38 × 0.25 mm

#### Data collection

Enraf–Nonius CAD-4 diffractometer
Absorption correction: ψ scan (North *et al.*, 1968 ▶) *T*_min_ = 0.591, *T*_max_ = 0.69
3153 measured reflections
2877 independent reflections
2644 reflections with *I* > 2σ(*I*)
*R*_int_ = 0.015
2 standard reflections every 100 reflections  intensity decay: 5%

#### Refinement

*R*[*F* ^2^ > 2σ(*F* ^2^)] = 0.023
*wR*(*F* ^2^) = 0.061
*S* = 1.07
2877 reflections
184 parameters
H atoms treated by a mixture of independent and constrained refinement
Δρ_max_ = 1.06 e Å^−3^
Δρ_min_ = −0.48 e Å^−3^

### 

Data collection: *CAD-4 EXPRESS* (Enraf–Nonius, 1994 ▶); cell refinement: *CAD-4 EXPRESS*; data reduction: *XCAD4* (Harms & Wocadlo, 1995 ▶); program(s) used to solve structure: *DIRDIF99* (Beurskens *et al.*, 1999 ▶); program(s) used to refine structure: *SHELXL97* (Sheldrick, 2008 ▶); molecular graphics: *ORTEP-3 for Windows* (Farrugia, 1997 ▶) and *PLATON* (Spek, 2009 ▶); software used to prepare material for publication: *WinGX* (Farrugia, 1999 ▶).

## Supplementary Material

Crystal structure: contains datablock(s) I, global. DOI: 10.1107/S1600536811022227/hb5901sup1.cif

Structure factors: contains datablock(s) I. DOI: 10.1107/S1600536811022227/hb5901Isup2.hkl

Additional supplementary materials:  crystallographic information; 3D view; checkCIF report

## Figures and Tables

**Table:** 

Ru1—C1	1.872 (3)
Ru1—C2	1.872 (2)
Ru1—N2	2.211 (2)
Ru1—N1	2.220 (2)
Ru1—Cl1	2.413 (2)
Ru1—Cl2	2.408 (2)

**Table:** 

N2—Ru1—N1	82.75 (9)

**Fig. 1.**
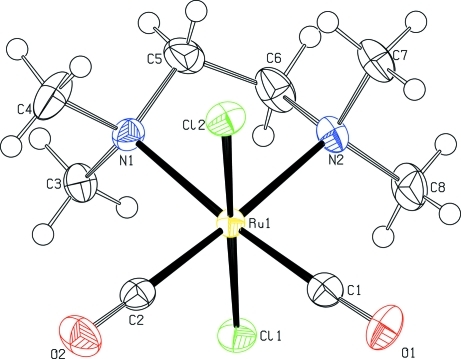
The molecular structure of (I) with displacement ellipsoids drawn at the 50% probability level.

**Fig. 2.**
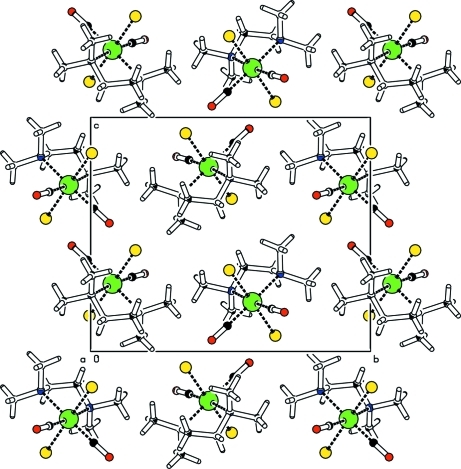
Unit cell packing for (I).

## supplementary crystallographic information

### Comment

The salt [Ru(CO)2Cl2]n was first reported by (Manchot & Konig, 1924) but its importance and chemistry was shown in late 1960's by (Stephenson & Wilkinson, 1966), (Kingston *et al.*, 1967) who have reported several compounds of the type [Ru(CO)2Cl2L2] where *L*=monodentate ligand. This was due to the fact that the salt [Ru(CO)2Cl2]n has proved a useful precursor for the synthesis of a variety of organometallic compounds. We have also reported from our laboratories compounds with ligands containing N, O and S atom as electron donor (e.g. Baghlaf *et al.*, 2007). However (Campbell (1975) and Padhey & Kaufman (1985) have reported about remarkable biological activities of such compounds against microbes, viruses and tumours. This has been the main reason for our research activity in the field of bio-inorganic chemistry of transition metal complexes. The metal atom Ru in the salt [Ru(CO)2Cl2]n being electron deficient acts as electron acceptor. This enhances its ability to coordinate with electron donor ligand (TMEDA) to give a stable octahedral electron rich compound of low ionization energy [Ru(CO)2Cl2TMEDA]. The bidentate nature of the ligand (TMEDA) has also been reported in the X-ray structure of the complex [Mo(CO)_4_TMEDA] by (Bakar *et al.*, 1993).

In the crystal structure of title compound, ruthenium atom is almost octahedrally coordinated to the two nitrogen-donors atoms of tetramethyl ethylene-1,2-diamine (TMEDA), two chloro and two carbonyl groups. A five membered non-planer ring formed through Ru1/N1/C5/C6/N2 as both of the carbon atoms are *sp*^3^ hybridized. The root mean square deniavtion for the ring measure 0.2149Å with the maximum deviation from C5 and C6 measures -0.2999 (19) Å and 0.3144 (19)Å respectively.

### Experimental

In a 100-ml round bottom flask fitted with nitrogen gas inlet, water condenser and magnetic stirrer was added 0.2 g of [Ru(CO)_2_ Cl_2_]n and 0.5 ml of tetramethylethylene diamine (TMEDA) in 15 ml MeOH. The reaction mixture was heated at about 70 °C for 1 h. The yellow green solution was reduced in volume and passed through a small alumina column (15 g. Al_2_O_3_). The yellow band was eluted with MeOH. The solvent was reduced in volume and on cooling it gave yellow blocks of (I). Yield 70%.

### Refinement

All the C—H H-atoms were positioned *via* fourier map with C—H = 0.91 (3)—1.11 (3) Å with *U*_iso_(H) = 1.2 *U*_eq_for aromatic C atoms.

### Crystal data

**Table:**

[RuCl_2_(C_6_H_16_N_2_)(CO)_2_]	*F*(000) = 688
*M**_r_* = 344.20	*D*_x_ = 1.722 Mg m^−^^3^
Monoclinic, *P*2_1_/*c*	Melting point: 493 K
Hall symbol: -P 2ybc	Mo *K*α radiation, λ = 0.71073 Å
*a* = 7.463 (6) Å	Cell parameters from 25 reflections
*b* = 14.579 (6) Å	θ = 10.6–14.0°
*c* = 12.718 (12) Å	µ = 1.57 mm^−^^1^
β = 106.37 (8)°	*T* = 160 K
*V* = 1327.7 (17) Å^3^	Block, yellow
*Z* = 4	0.38 × 0.38 × 0.25 mm

### Data collection

**Table:**

Enraf–Nonius CAD-4 diffractometer	2644 reflections with *I* > 2σ(*I*)
Radiation source: fine-focus sealed tube	*R*_int_ = 0.015
graphite	θ_max_ = 27.0°, θ_min_ = 2.2°
Nonprofiled ω/2θ scans	*h* = −9→9
Absorption correction: ψ scan (North *et al.*, 1968)	*k* = 0→18
*T*_min_ = 0.591, *T*_max_ = 0.69	*l* = 0→16
3153 measured reflections	2 standard reflections every 100 reflections
2877 independent reflections	intensity decay: 5%

### Refinement

**Table:**

Refinement on *F*^2^	Primary atom site location: structure-invariant direct methods
Least-squares matrix: full	Secondary atom site location: difference Fourier map
*R*[*F*^2^ > 2σ(*F*^2^)] = 0.023	Hydrogen site location: inferred from neighbouring sites
*wR*(*F*^2^) = 0.061	H atoms treated by a mixture of independent and constrained refinement
*S* = 1.07	*w* = 1/[σ^2^(*F*_o_^2^) + (0.0385*P*)^2^ + 0.6002*P*] where *P* = (*F*_o_^2^ + 2*F*_c_^2^)/3
2877 reflections	(Δ/σ)_max_ = 0.001
184 parameters	Δρ_max_ = 1.06 e Å^−^^3^
0 restraints	Δρ_min_ = −0.48 e Å^−^^3^

### Special details

**Table:**

Geometry. All s.u.'s (except the s.u. in the dihedral angle between two l.s. planes) are estimated using the full covariance matrix. The cell s.u.'s are taken into account individually in the estimation of s.u.'s in distances, angles and torsion angles; correlations between s.u.'s in cell parameters are only used when they are defined by crystal symmetry. An approximate (isotropic) treatment of cell s.u.'s is used for estimating s.u.'s involving l.s. planes.
Refinement. Refinement of *F*^2^ against ALL reflections. The weighted *R*-factor *wR* and goodness of fit *S* are based on *F*^2^, conventional *R*-factors *R* are based on *F*, with *F* set to zero for negative *F*^2^. The threshold expression of *F*^2^ > σ(*F*^2^) is used only for calculating *R*-factors(gt) *etc*. and is not relevant to the choice of reflections for refinement. *R*-factors based on *F*^2^ are statistically about twice as large as those based on *F*, and *R*- factors based on ALL data will be even larger.

### Fractional atomic coordinates and isotropic or equivalent isotropic displacement parameters (Å^2^)

**Table:**

	*x*	*y*	*z*	*U*_iso_*/*U*_eq_	
C1	−0.0497 (3)	0.14822 (15)	0.31835 (17)	0.0244 (4)	
C2	0.1219 (3)	−0.01145 (15)	0.39077 (17)	0.0237 (4)	
C3	0.5479 (3)	0.00447 (17)	0.3248 (2)	0.0269 (4)	
H3A	0.634 (4)	−0.025 (2)	0.292 (2)	0.032*	
H3B	0.542 (4)	−0.025 (2)	0.386 (2)	0.032*	
H3C	0.587 (4)	0.065 (2)	0.342 (2)	0.032*	
C4	0.3204 (4)	−0.09661 (19)	0.2146 (3)	0.0397 (6)	
H4A	0.197 (5)	−0.105 (2)	0.163 (3)	0.048*	
H4B	0.331 (4)	−0.131 (2)	0.294 (3)	0.048*	
H4C	0.417 (5)	−0.121 (2)	0.187 (3)	0.048*	
C5	0.3756 (4)	0.0491 (2)	0.1397 (2)	0.0376 (6)	
H5A	0.269 (5)	0.028 (2)	0.078 (3)	0.045*	
H5B	0.496 (5)	0.032 (2)	0.127 (3)	0.045*	
C6	0.3660 (4)	0.1513 (2)	0.1516 (2)	0.0362 (6)	
H6A	0.459 (5)	0.170 (2)	0.218 (3)	0.043*	
H6B	0.373 (4)	0.180 (2)	0.087 (3)	0.043*	
C7	0.0379 (4)	0.17649 (19)	0.0602 (2)	0.0352 (5)	
H7A	−0.077 (5)	0.197 (2)	0.078 (2)	0.042*	
H7B	0.026 (4)	0.115 (2)	0.023 (3)	0.042*	
H7C	0.077 (4)	0.219 (2)	0.010 (3)	0.042*	
C8	0.1967 (4)	0.27503 (17)	0.2056 (2)	0.0385 (6)	
H8A	0.229 (5)	0.311 (2)	0.152 (3)	0.046*	
H8B	0.285 (5)	0.285 (2)	0.277 (3)	0.046*	
H8C	0.075 (5)	0.294 (2)	0.214 (2)	0.046*	
Cl1	0.36315 (7)	0.16019 (4)	0.43227 (4)	0.02799 (12)	
Cl2	−0.09471 (7)	−0.00421 (4)	0.15266 (4)	0.02621 (12)	
O1	−0.1658 (2)	0.19233 (12)	0.33235 (15)	0.0343 (4)	
O2	0.1117 (3)	−0.06675 (12)	0.45157 (15)	0.0377 (4)	
Ru1	0.14044 (2)	0.078019 (10)	0.288724 (12)	0.01683 (7)	
N1	0.3635 (2)	0.00254 (13)	0.24068 (15)	0.0233 (4)	
N2	0.1815 (3)	0.17845 (13)	0.16732 (15)	0.0246 (4)	

### Atomic displacement parameters (Å^2^)

**Table:**

	*U*^11^	*U*^22^	*U*^33^	*U*^12^	*U*^13^	*U*^23^
C1	0.0238 (10)	0.0231 (10)	0.0239 (10)	−0.0067 (9)	0.0027 (8)	−0.0024 (8)
C2	0.0224 (10)	0.0243 (10)	0.0224 (9)	−0.0033 (8)	0.0034 (8)	−0.0050 (8)
C3	0.0185 (10)	0.0303 (12)	0.0289 (11)	0.0033 (9)	0.0018 (8)	0.0029 (9)
C4	0.0287 (12)	0.0310 (13)	0.0561 (18)	0.0060 (10)	0.0064 (12)	−0.0169 (12)
C5	0.0329 (13)	0.0558 (16)	0.0263 (12)	0.0170 (12)	0.0120 (10)	0.0091 (11)
C6	0.0270 (12)	0.0467 (15)	0.0368 (13)	0.0019 (11)	0.0119 (10)	0.0174 (12)
C7	0.0338 (13)	0.0380 (14)	0.0281 (12)	0.0010 (11)	−0.0007 (10)	0.0110 (10)
C8	0.0492 (16)	0.0200 (11)	0.0423 (14)	−0.0074 (11)	0.0065 (12)	0.0060 (10)
Cl1	0.0275 (3)	0.0288 (3)	0.0241 (2)	−0.0044 (2)	0.0016 (2)	−0.0063 (2)
Cl2	0.0198 (2)	0.0304 (3)	0.0266 (2)	−0.00546 (19)	0.00355 (19)	−0.0077 (2)
O1	0.0267 (8)	0.0290 (9)	0.0485 (10)	0.0042 (7)	0.0129 (7)	−0.0069 (7)
O2	0.0473 (11)	0.0342 (9)	0.0326 (9)	−0.0072 (8)	0.0129 (8)	0.0088 (8)
Ru1	0.01583 (10)	0.01612 (10)	0.01779 (10)	−0.00122 (5)	0.00351 (7)	−0.00079 (5)
N1	0.0207 (8)	0.0256 (9)	0.0229 (8)	0.0035 (7)	0.0049 (7)	−0.0021 (7)
N2	0.0242 (9)	0.0230 (9)	0.0244 (9)	−0.0019 (7)	0.0035 (7)	0.0046 (7)

### Geometric parameters (Å, °)

**Table:**

Ru1—C1	1.872 (3)	C5—N1	1.477 (3)
Ru1—C2	1.872 (2)	C5—C6	1.502 (4)
Ru1—N2	2.211 (2)	C5—H5A	1.00 (3)
Ru1—N1	2.220 (2)	C5—H5B	0.99 (3)
Ru1—Cl1	2.413 (2)	C6—N2	1.500 (3)
Ru1—Cl2	2.408 (2)	C6—H6A	0.97 (3)
C1—O1	1.133 (3)	C6—H6B	0.94 (3)
C2—O2	1.134 (3)	C7—N2	1.477 (3)
C3—N1	1.486 (3)	C7—H7A	1.00 (3)
C3—H3A	0.96 (3)	C7—H7B	1.00 (3)
C3—H3B	0.91 (3)	C7—H7C	0.99 (3)
C3—H3C	0.93 (3)	C8—N2	1.484 (3)
C4—N1	1.498 (3)	C8—H8A	0.94 (3)
C4—H4A	0.98 (4)	C8—H8B	0.97 (3)
C4—H4B	1.11 (3)	C8—H8C	0.98 (3)
C4—H4C	0.95 (4)		
			
O1—C1—Ru1	177.3 (2)	N2—C8—H8B	114.7 (19)
O2—C2—Ru1	178.8 (2)	H8A—C8—H8B	110 (3)
N1—C3—H3A	106.0 (16)	N2—C8—H8C	108.2 (19)
N1—C3—H3B	110.6 (18)	H8A—C8—H8C	111 (3)
H3A—C3—H3B	111 (2)	H8B—C8—H8C	105 (3)
N1—C3—H3C	110.5 (19)	C1—Ru1—C2	91.90 (11)
H3A—C3—H3C	109 (3)	C1—Ru1—N2	92.33 (10)
H3B—C3—H3C	110 (3)	C2—Ru1—N2	175.71 (8)
N1—C4—H4A	111.8 (19)	C1—Ru1—N1	174.94 (8)
N1—C4—H4B	106.3 (17)	C2—Ru1—N1	93.04 (10)
H4A—C4—H4B	111 (3)	N2—Ru1—N1	82.75 (9)
N1—C4—H4C	108 (2)	C1—Ru1—Cl2	88.66 (9)
H4A—C4—H4C	112 (3)	C2—Ru1—Cl2	88.29 (9)
H4B—C4—H4C	107 (3)	N2—Ru1—Cl2	92.53 (8)
N1—C5—C6	110.6 (2)	N1—Ru1—Cl2	90.38 (8)
N1—C5—H5A	108.2 (18)	C1—Ru1—Cl1	88.62 (9)
C6—C5—H5A	109.8 (18)	C2—Ru1—Cl1	89.49 (9)
N1—C5—H5B	108.0 (18)	N2—Ru1—Cl1	89.89 (8)
C6—C5—H5B	110.0 (19)	N1—Ru1—Cl1	92.53 (8)
H5A—C5—H5B	110 (3)	Cl2—Ru1—Cl1	176.424 (19)
N2—C6—C5	110.3 (2)	C5—N1—C3	110.3 (2)
N2—C6—H6A	105.4 (18)	C5—N1—C4	108.2 (2)
C5—C6—H6A	108.6 (18)	C3—N1—C4	105.97 (18)
N2—C6—H6B	106.0 (19)	C5—N1—Ru1	104.24 (15)
C5—C6—H6B	110.2 (19)	C3—N1—Ru1	113.96 (15)
H6A—C6—H6B	116 (3)	C4—N1—Ru1	114.10 (15)
N2—C7—H7A	103.4 (18)	C7—N2—C8	106.8 (2)
N2—C7—H7B	113.4 (18)	C7—N2—C6	109.1 (2)
H7A—C7—H7B	114 (3)	C8—N2—C6	107.8 (2)
N2—C7—H7C	108.3 (19)	C7—N2—Ru1	115.10 (15)
H7A—C7—H7C	113 (2)	C8—N2—Ru1	114.35 (16)
H7B—C7—H7C	105 (2)	C6—N2—Ru1	103.38 (14)
N2—C8—H8A	107.7 (19)		
			
N1—C5—C6—N2	−62.9 (3)	C5—C6—N2—C7	−78.1 (2)
C6—C5—N1—C3	−80.0 (2)	C5—C6—N2—C8	166.3 (2)
C6—C5—N1—C4	164.5 (2)	C5—C6—N2—Ru1	44.8 (2)
C6—C5—N1—Ru1	42.7 (2)	C1—Ru1—N2—C7	−76.26 (19)
C1—Ru1—N1—C5	−0.1 (9)	C2—Ru1—N2—C7	113.4 (10)
C2—Ru1—N1—C5	167.17 (16)	N1—Ru1—N2—C7	102.56 (18)
N2—Ru1—N1—C5	−13.63 (16)	Cl2—Ru1—N2—C7	12.49 (17)
Cl2—Ru1—N1—C5	78.87 (17)	Cl1—Ru1—N2—C7	−164.88 (17)
Cl1—Ru1—N1—C5	−103.20 (17)	C1—Ru1—N2—C8	48.00 (19)
C1—Ru1—N1—C3	120.1 (9)	C2—Ru1—N2—C8	−122.3 (10)
C2—Ru1—N1—C3	−72.57 (17)	N1—Ru1—N2—C8	−133.18 (18)
N2—Ru1—N1—C3	106.62 (16)	Cl2—Ru1—N2—C8	136.76 (17)
Cl2—Ru1—N1—C3	−160.87 (15)	Cl1—Ru1—N2—C8	−40.61 (17)
Cl1—Ru1—N1—C3	17.05 (15)	C1—Ru1—N2—C6	164.90 (16)
C1—Ru1—N1—C4	−117.9 (9)	C2—Ru1—N2—C6	−5.4 (11)
C2—Ru1—N1—C4	49.35 (19)	N1—Ru1—N2—C6	−16.28 (15)
N2—Ru1—N1—C4	−131.46 (18)	Cl2—Ru1—N2—C6	−106.34 (16)
Cl2—Ru1—N1—C4	−38.96 (17)	Cl1—Ru1—N2—C6	76.29 (16)
Cl1—Ru1—N1—C4	138.97 (17)		
